# Association between adverse childhood experiences and over‐the‐counter drug abuse in Japan: A nationwide population‐based cross‐sectional study

**DOI:** 10.1002/pcn5.70354

**Published:** 2026-06-02

**Authors:** Yuhei Mori, Takashi Yoshioka, Risa Shishido, Mizuki Hino, Akiko Sato, Atsuko Nagaoka, Masataka Hatano, Yuto Hosogai, Kazusa Miyahara, Takahiro Tabuchi, Itaru Miura, Yasuto Kunii

**Affiliations:** ^1^ Department of Neuropsychiatry School of Medicine Fukushima Medical University Fukushima Japan; ^2^ Shizuoka Graduate University of Public Health Shizuoka Japan; ^3^ Department of Disaster Psychiatry International Research Institute of Disaster Science Tohoku University Sendai Japan; ^4^ Department of Psychiatry Graduate School of Medicine Tohoku University Miyagi Japan; ^5^ Division of Epidemiology School of Public Health, Graduate School of Medicine Tohoku University Miyagi Japan

**Keywords:** addiction, adverse childhood experiences, drug abuse, epidemiological study, over‐the‐counter medications

## Abstract

**Aim:**

We aimed to examine the association between adverse childhood experiences (ACEs) and habitual over‐the‐counter (OTC) drug abuse in a nationwide Japanese population and determine whether this association varies with other substance use behaviors.

**Methods:**

We analyzed nationwide cross‐sectional data and used multivariable logistic regression to estimate adjusted odds ratios (aORs) for the association between ACEs and OTC drug abuse. We also examined whether this association varied by other addictive behaviors, including habitual use of combustible cigarettes, electronic cigarettes (e‐cigarettes), heated tobacco products (HTPs), alcohol, or illicit drugs.

**Results:**

We analyzed 25,424 adults from the 2025 Japan Society and New Tobacco Internet Survey (Feb 23 to Mar 31, 2025). Habitual OTC drug abuse prevalence was 6.8% (95% confidence interval [CI]: 6.2–7.4). Individuals with high ACE exposure had significantly higher odds of OTC drug abuse than those with lower ACE exposure (aOR = 3.18, 95% CI: 2.47–4.10, *p* < 0.001). The association was particularly strong among users of e‐cigarettes (aOR = 27.14, 95% CI: 13.12–56.14) and HTPs (aOR = 6.88, 95% CI: 4.21–11.24). However, no significant interaction was observed among participants reporting illicit drug use (*p* = 0.14).

**Conclusion:**

ACEs were independently associated with habitual OTC drug abuse in this study. Similar associations were observed among individuals with other addictive behaviors, with higher odds indicating potential overlap in vulnerability pathways. These findings underscore the lasting impact of childhood adversity and the need for trauma‐informed strategies to prevent emerging substance abuse.

## INTRODUCTION

Adverse childhood experiences (ACEs) have attracted increasing attention in psychiatry and public health from the perspective of life‐course epidemiology.^1^, ^2^, ^3^, ^4^ They include a wide range of childhood adversities, such as physical, sexual, and emotional abuse; neglect; exposure to domestic violence; and family dysfunction. These early‐life adversities have long‐term effects on physical and mental health and increase vulnerability to mood and substance use disorders.^5^, ^6^, ^7^ A large‐scale cohort study in Japan found that approximately three‐quarters of adults had experienced at least one ACE,^8^ suggesting that childhood adversity is a widespread public health issue rather than an exceptional condition.

Over‐the‐counter (OTC) drug abuse has become a growing social and medical concern in Japan.^9^, ^10^, ^11^ A nationwide survey by the Ministry of Health, Labour and Welfare reported that approximately 1.8% of junior high school students (approximately 1 in 55) had abused OTC drugs to alter their mood or achieve euphoria within the past year. This proportion exceeded the prevalence of any illicit drug use in the same population.^12^ The report also indicated that adolescents with a history of OTC drug abuse often lived in social isolation at school and home and experienced various forms of psychological and social distress in their everyday lives.

Evidence suggests that psychosocial stress and emotional distress are associated with OTC drug abuse. For example, clinical and epidemiological studies have reported that individuals who abuse OTC drugs often present with psychiatric comorbidities, repeated overdoses, and dependence‐like patterns, and that emotional strain during the COVID‐19 pandemic increased vulnerability among at‐risk individuals.^13^, ^14^, ^15^, ^16^ Prior studies have also shown that ACEs are associated with a wide range of substance use outcomes.^5^, ^6^, ^7^

ACEs may be associated with OTC drug abuse through psychosocial and emotional distress, given that ACEs can contribute to persistent psychological distress, impaired emotion regulation, and maladaptive coping strategies, and that psychosocial distress has been linked to OTC drug abuse. Notably, OTC medications may be particularly relevant in this pathway because they are legally available, easily accessible through pharmacies and online retailers, and less stigmatized than illicit substances, making them especially susceptible to abuse among adolescents and young adults.^17^ In Japan, clinical and epidemiological studies have also reported that OTC drug abuse is associated with psychosocial difficulties, including school and home isolation, as well as psychiatric comorbidity.^15^, ^18^ However, despite this plausible link, empirical evidence directly examining the association between ACEs and OTC drug abuse remains scarce.

Clarifying this association is essential for early intervention and prevention. Therefore, in this study, we aimed to examine the association between ACEs and OTC drug abuse in a nationwide Japanese sample.

## METHODS

### Ethical considerations

All procedures were conducted in accordance with the ethical guidelines of the Declaration of Helsinki and Japan's Ethical Guidelines for Medical and Health Research Involving Human Participants. The study was approved by the ethics committees of Fukushima Medical University (2025‐002) and Tohoku University Graduate School of Medicine (2024‐1‐673).

### Participants

**Figure 1:**
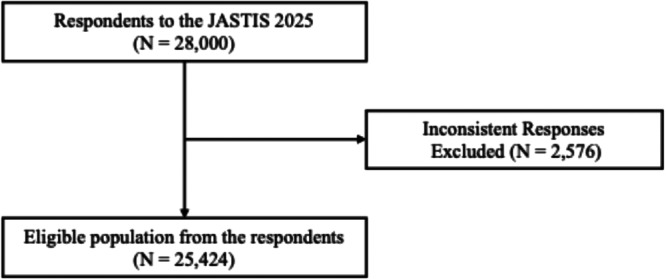
Flowchart of the study population. *Note*: JASTIS, Japan Society and New Tobacco Internet Survey.

The data used in this study were sourced from the 2025 Japan Society and New Tobacco Internet Survey (JASTIS)^19^ conducted by Rakuten Insight, Inc. (Tokyo, Japan) from February 23 to March 31, 2025. The study design and protocol have been detailed previously.^20^, ^21^ Participants were recruited from the survey panel of Rakuten Insight, Inc., a large internet research company in Japan with approximately 2.2 million registered panelists. Invitations were distributed using quota sampling based on sex, age, and prefecture to approximate the demographic distribution of the Japanese population. Participants received “E‐points,” which could be redeemed for online purchases or cash, as compensation for their participation. The amount of E‐points provided to respondents as an incentive was not disclosed to the researchers. The survey targeted a representative sample of 28,000 participants to reflect diverse demographic and socioeconomic distributions. Of the 28,000 JASTIS 2025 respondents, 2576 were excluded based on the following criteria: short response time (less than 600 s), failure to pass an attention‐check question, reporting of all listed drug use categories, listing of all health conditions, and reporting of more than 15 family members. Consequently, data from 25,424 participants were used for the analysis (Figure 1).

### ACEs

ACEs were assessed using the Japanese version of the ACE Study questionnaire, which was translated and psychometrically validated by Tsuboi as part of a Grant‐in‐Aid project (KAKENHI 24790625)^22^ based on the original Centers for Disease Control and Prevention (CDC)‐Kaiser ACE Study questionnaire.^23^ The research team granted explicit permission for its use in the present study. Participants were instructed to retrospectively indicate whether they had experienced any of the 10 ACEs before they reached 18 years of age. Each item was coded as a binary variable (yes or no), and the total ACE score was calculated by summing the number of endorsed items, yielding a score ranging from 0 to 10. The 10 ACE items are listed in Supporting Information S1: Table 1. High ACE exposure was defined as four or more ACEs, consistent with previous epidemiological studies that have widely used this cutoff as an indicator of high cumulative adversity.^4^, ^5^ However, this cutoff has not been specifically validated for the Japanese version used in the present study.

### Outcome variables

#### Primary outcome variables

The primary outcome of this study was current OTC drug abuse, which was identified based on the responses to a three‐step nested questionnaire that assessed (1) use, (2) misuse, and (3) abuse within the past 12 months. Participants were first asked whether they had used any OTC medications on five or more occasions during the past year (“use”). Those who responded affirmatively were asked whether they had ever taken any OTC medications in quantities exceeding the recommended dose (“misuse”). They were subsequently asked whether they had used OTC medications for non‐medical or euphoric purposes, defined as taking them “to get high or to change one's mood beyond the therapeutic dose” (“abuse”). Participants who met all three criteria (use, misuse, and abuse) were classified as current abusers of OTC drugs. The survey covered several commonly available OTC medications in Japan, including cold medicines, cough suppressants/expectorants, analgesics and anti‐inflammatory agents, antihistamines/anti‐allergy drugs, sedatives, sleep aids, and caffeine‐containing stimulants.

#### Covariates and additional measures

Covariates included age, sex, marital status, educational attainment, employment status, household income, living arrangement, psychological distress measured using the Kessler‐6 (K6) scale,^24^ and loneliness measured using a short‐form 3 items of the University of California Los Angeles Loneliness Scale version 3 (UCLA‐SF3).^25^ We used Question 1 of the Alcohol Use Disorders Identification Test to assess alcohol consumption.^26^ Participants were asked, “How often do you have a drink containing alcohol?” responses of “2–3 times a week” or “4 or more times a week” were categorized as habitual alcohol use. The K6 consists of six items rated on a 5‐point scale (0–4), yielding a total score ranging from 0 to 24. Higher scores indicate greater psychological distress. The K6 scores were treated as continuous variables and dichotomized using a cutoff of ≥13 to identify severe psychological distress, following established thresholds.^27^, ^28^ Loneliness was dichotomized using the median UCLA‐SF3 score, and the participants were categorized into high (≥6) and low (<6) groups. This approach was chosen because no established clinical cutoff exists for the UCLA‐SF3, and median‐based categorization has been commonly employed in prior epidemiological studies.

### Statistical analysis

We conducted a cross‐sectional analysis of data from the JASTIS 2025 study to examine the association between ACEs and OTC drug abuse.

#### Main analysis

The primary logistic regression model used OTC drug abuse (yes/no) as the dependent variable and high ACE exposure (≥4 vs. ≤3 ACEs) as the main independent variable. As additional analyses, we repeated the logistic regression models using the total ACE score recalculated from the 10 binary ACE items as a continuous variable and as an ordinal categorical variable, with a score of 0 as the reference category. Covariates were selected a priori based on previous studies and included age, sex, educational attainment, marital status, employment status, household income (including a fourth‐degree polynomial), combustible cigarette use, electronic cigarette (e‐cigarette) use, alcohol use, illicit drug use, heated tobacco products (HTPs) use, psychological distress (K6 score), and loneliness (UCLA‐SF3 score). The variables were categorized as follows: sex (male, female); age (15–19, 20–29, 30–39, 40–49, 50–64, and ≥65 years); marital status (married, single/divorced); living arrangement (lives alone, lives with others); educational attainment (less than high school/high school, college, undergraduate, graduate or higher, and unknown); household income (<4 million yen, 4–6 million yen, 6–8 million yen, 8–10 million yen, and ≥10 million yen); and employment status (paid work, no paid work, and students).

#### Interaction analysis

To examine whether the association between ACEs and OTC drug abuse differed across subgroups, we incorporated multiplicative interaction terms into the logistic regression models. Interaction terms between ACEs and each substance use variable (alcohol, combustible cigarette, e‐cigarette, HTPs, and illicit drug use) were tested separately. A likelihood ratio test was used to assess the statistical significance of the interaction, and the Benjamini–Hochberg method was applied to control the false discovery rate for multiple comparisons.

#### Stratified analysis

Stratified logistic regression analyses were also conducted for each subgroup of the moderators with potential interaction effects. The adjusted odds ratios (ORs) and 95% confidence intervals (CIs) for the association between ACEs and OTC drug abuse were determined for users and non‐users of each substance. These results were summarized to highlight the variations in the strength of the ACE–OTC drug abuse association across the behavioral risk profiles.

In all analyses, inverse probability weighting based on demographic distributions was applied to adjust for potential sampling bias in the internet‐based survey. Statistical analyses were performed using spss version 30.0.0.0 and R software (R Foundation for Statistical Computing, Vienna, Austria). Statistical significance was set at *p* < 0.05.

## RESULTS

### Characteristics of the participants

Table 1 presents demographic characteristics of the participants stratified by their ACE scores (≥4 vs. <4). The prevalence of each ACE item and the distribution of total ACE scores are shown in Supporting Information S2: Table 2. Overall, 26.2% of participants reported at least one ACE, and 5.4% had high ACE exposure, defined as four or more ACEs. Of the 25,424 participants, 6.8% reported OTC drug abuse (Table 2). Of the 25,424 respondents, 23,927 with complete data on exposure, outcome, covariates, and sampling weights were included in the multivariable and subgroup analyses. Multivariable logistic regression revealed a significant association between ACEs and a higher likelihood of habitual OTC drug abuse (OR = 3.18, 95% CI = 2.47–4.10, *p* < 0.001) (Table 3). In additional analyses, the total ACE score recalculated from the 10 ACE items was significantly associated with OTC drug abuse when modeled as a continuous variable (OR per 1‐point increase = 1.26, 95% CI = 1.20–1.33, *p* < 0.001). Ordinal categorical analyses also showed a graded increase in the odds of OTC drug abuse with increasing ACE scores, although estimates for the highest scores should be interpreted cautiously because of small cell counts (Supporting Information S3: Table 3).

**Table 1:** Characteristics of participants (*N* = 25,424) stratified by adverse childhood experience (ACE) score (≥4 vs. <4).

Variable	High ACE group (*n* = 1377)		Non‐high ACE group (*n* = 24,047)	
	*n*	%	*n*	%
Sex				
Male	642	46.6	12,069	50.2
Female	735	53.4	11,978	49.8
Age				
15–19 years old	30	2.2	341	1.4
20–29 years old	340	24.7	3663	15.2
30–39 years old	281	20.4	3999	16.6
40–49 years old	271	19.7	4040	16.8
50–64 years old	354	25.7	6230	25.9
65 or older	101	7.3	5774	24.0
Marital status				
Married	651	47.3	14,302	59.5
Single/divorced	726	52.7	9745	40.5
Living arrangement				
Lives alone	306	22.2	4150	17.3
Lives with others	1071	77.8	19,897	82.7
Educational attainment				
Less than high school or high school	421	30.6	6153	25.6
College	278	20.2	5019	20.9
Undergraduate	523	38.0	10,541	43.8
Graduate over	66	4.8	1284	5.3
unknown	89	6.5	1050	4.4
Household income				
<4 million yen	542	39.4	9027	37.5
4–6 million yen	318	23.1	5735	23.8
6–8 million yen	192	13.9	3969	16.5
8–10 million yen	169	12.3	2525	10.5
Over 10 million yen	156	11.3	2791	11.6
Work				
Paid work	1022	74.2	16,701	69.5
No paid work	279	20.3	6421	26.7
Students	76	5.5	925	3.8

**Table 2:** Prevalence of habitual over‐the‐counter drug abuse stratified by age group.

Participants	Prevalence: % (95% CI)
15–19 years old	13.6 (3.6–23.6)
20–29 years old	10.4 (8.6–12.2)
30–39 years old	7.5 (6.1–8.8)
40–49 years old	6.8 (5.5–8.0)
50–64 years old	5.3 (4.3–6.2)
65 years old or older	5.1 (3.9–6.3)
All participants	6.8 (6.2–7.4)

Abbreviation: CI, confidence interval.

**Table 3:** Results of multivariable logistic regression analyses examining associations between adverse childhood experience (ACE) and other covariates with over‐the‐counter (OTC) drug abuse.

Variable	OR	95% CI	*p*‐Value
ACE	3.18	2.47–4.10	<0.001
Sex (female)	0.89	0.72–1.10	0.28
Age	0.99	0.98–1.00	0.10
Education (L)	0.86	0.46–1.63	0.65
Education (Q)	0.88	0.50–1.54	0.65
Education (C)	0.99	0.66–1.47	0.96
Education (^4)	1.04	0.83–1.32	0.73
Marriage (never)	0.76	0.58–0.99	0.04
Family (with others)	1.10	0.80–1.51	0.55
Alcohol (yes)	0.95	0.76–1.18	0.61
Combustible cigarette (yes)	0.99	0.76–1.29	0.92
Electronic cigarette (yes)	3.35	2.37–4.73	<0.001
Heated tobacco smoking products (yes)	2.26	1.70–2.99	<0.001
Illicit drug (yes)	5.97	3.81–9.36	<0.001
High K6 group (≥13)	1.52	1.17–1.98	0.002
UCLA Loneliness Scale (short‐form 3 items)	1.35	1.05–1.73	0.02
Income (L)	0.79	0.59–1.08	0.14
Income (Q)	1.37	1.04–1.80	0.02
Income (C)	0.91	0.71–1.16	0.43
Income (^4)	1.23	0.98–1.54	0.08
Job (no paid work)	0.76	0.57–1.00	0.05
Job (students)	1.95	0.62–6.08	0.25

*Note*: Variable explanations—ACE: adverse childhood experience. Sex: sex (female vs. male). Age: age (continuous). Education.L, Education.Q, Education.C, and Education^4: orthogonal polynomial contrasts representing linear (L), quadratic (Q), cubic (C), and quartic trends, respectively, for educational level. The original categories of education were: less than high school or high school, college, undergraduate, graduate and above, and unknown. Marriage: marital status (never vs. married). Family: living arrangement (lives_with_other vs. lives_alone). Alcohol: alcohol use (yes vs. no). K6: psychological distress (binary; ≥13 vs. ≤12). UCLA: loneliness score (binary; ≥6 vs. ≤5). Income.L, Income.Q, Income.C, and Income^4: orthogonal polynomial terms for income categories allow for the assessment of linear and non‐linear trends across ordinal income levels (original categories: <4 million yen, 4–6 million yen, 6–8 million yen, 8–10 million yen, and >10 million yen). Specifically, these terms capture whether increases in income are associated with monotonic increases or decreases in the outcome (linear), or more complex patterns such as U‐shaped or cubic trends (quadratic or cubic). Job: employment status (paid_work, no_paid_work, and students). “Yes” indicates presence or absence of each addictive behavior.

Abbreviations: CI, confidence interval; K6, Kessler Psychological Distress Scale; OR, odds ratio; UCLA‐LS3 (SF‐3), UCLA‐SF3, three‐item short form of the UCLA Loneliness Scale.

### Interaction analysis

Interaction terms were introduced into the model to test whether the effect of ACEs on OTC drug abuse varied by other substance use behaviors (Table 4A). Significant interactions were observed between ACEs and the use of HTPs (Benjamini–Hochberg correction [BH]‐adjusted *p* < 0.001), combustible cigarettes (BH‐adjusted *p* = 0.014), and e‐cigarettes (BH‐adjusted *p* = 1.2 × 10^−8^). The interaction with illicit drug use was not statistically significant (*p* = 0.14). Stronger associations between ACEs and OTC drug abuse were observed for participants who used HTPs, combustible cigarettes, or e‐cigarettes than for those who did not (e‐cigarette users: OR = 27.1, 95% CI = 13.1–56.1 vs. non‐users: OR = 2.40, 95% CI = 1.76–3.27).

**Table 4:** Interaction and stratified analyses of the association between adverse childhood experiences and habitual over‐the‐counter drug abuse across subgroups of addictive behaviors.

A: interaction analysis					
Moderator	Level	Adjusted OR (ACE)	95% CI	*p* for interaction	Adjusted *p* (BH)
Alcohol use	Yes	3.82	2.47–5.91	0.36	0.36
Alcohol use	No	2.98	2.19–4.05	0.36	0.36
HTPs use	Yes	6.88	4.21–11.24	<0.001	<0.001
HTPs use	No	2.17	1.54–3.06	<0.001	<0.001
Combustible cigarette use	Yes	6.33	3.60–11.25	0.01	0.01
Combustible cigarette use	No	2.67	2.00–3.57	0.01	0.01
Electronic cigarette use	Yes	27.13	13.18–56.14	<0.0001	<0.0001
Electronic cigarette use	No	2.40	1.76–3.27	<0.0001	<0.0001
Illicit drug use	Yes	1.66	0.68–4.08	0.14	0.18
Illicit drug use	No	3.35	2.58–4.33	0.14	0.18

B: stratified analysis					
Moderator	Level	*n*	Events	Adjusted OR (ACE)	95% CI
Alcohol use	Yes	7326	483	3.82	2.51–5.82
Alcohol use	No	16,601	1000	3.00	2.21–4.08
HTPs use	Yes	2493	480	5.95	3.58–9.90
HTPs use	No	21,434	1003	2.21	1.56–3.13
Cigarette use	Yes	2765	321	5.39	3.15–9.22
Cigarette use	No	21,162	1162	2.70	2.10–3.62
Electronic cigarette use	Yes	631	259	20.26	10.24–40.04
Electronic cigarette use	No	23,296	1224	2.41	1.77–3.30
Illicit drug use	Yes	475	183	2.51	1.01–6.22
Illicit drug use	No	23,452	1300	3.30	2.55–4.28

*Note*: “Yes” and “No” indicate presence or absence of each addictive behavior (such as current use of alcohol, cigarette, or e‐cigarette).

Abbreviations: ACE, adverse childhood experience; BH, Benjamini–Hochberg correction; CI, confidence interval; HTPs, heated tobacco products; OR, odds ratio.

### Stratified analysis

We performed stratified logistic regression analyses based on the substance use category to further illustrate these interactions (Table 4B). The adjusted ORs for the prediction of OTC drug abuse by ACEs were 5.95 (95% CI = 3.58–9.90) and 2.21 (95% CI = 1.56–3.13) for HTP users and non‐users, respectively. The corresponding ORs for combustible cigarette users and non‐users were 5.39 (95% CI = 3.15–9.22) and 2.70 (95% CI = 2.01–3.62), respectively.

The strongest association was observed for e‐cigarette users (OR = 20.25, 95% CI = 10.24–40.04). No significant differences were observed across the alcohol and illicit drug use strata.

## DISCUSSION

In this nationwide population‐based analysis, we found that individuals with high ACE exposure (four or more ACEs) exhibited more than three times the odds of abusing OTC drugs than those with fewer than four ACEs. This association remained consistent when the total ACE score was analyzed as a continuous and ordinal categorical variable, suggesting that the finding was not solely dependent on the dichotomized cutoff. Interaction and stratified analyses also indicated variability in the association across subgroups, with higher odds observed among users of e‐cigarettes and combustible cigarettes, while no notable interaction emerged for illicit drug use.

This finding builds on previous evidence of the association between ACEs and various forms of substance use, such as cigarette smoking, harmful alcohol consumption, and illicit drug use.^7^, ^29^ It also suggests that early‐life adversity may increase vulnerability to the abuse of medications that are otherwise legally available. Recent evidence from meta‐analysis highlights the breadth of these associations across substance classes and supports the generalized vulnerability model of addiction risk.^30^

Subgroup and interaction analyses provided insight into the heterogeneity of the ACE–OTC drug abuse association. The association was pronounced among habitual users of e‐cigarettes or HTPs, which supports the notion that multiple addictive behaviors may share common psychosocial and neurobiological pathways. The “common liability to addictions” framework posits a non–drug‐specific liability that cuts across substances and behaviors and offers a coherent account of why clustering of addictions is frequently observed in epidemiologic data.^31^ HTPs have spread rapidly in Japan, particularly among younger adults. Their prevalence of current use was approximately 11% in 2020, which provides relevant context for the interaction between ACEs and HTP use in our analysis.^32^ However, the large odds ratios observed among e‐cigarette and HTP users should be interpreted cautiously. These estimates may partly reflect statistical instability due to the relatively small number of participants and events in some subgroups, as well as sparse data in interaction and stratified analyses.^33^ Therefore, these findings should be regarded as suggestive of possible effect modification rather than as precise estimates of the magnitude of association. Differences in nicotine delivery, sensory effects, and patterns of use across combustible cigarettes, e‐cigarettes, and HTPs may also partly contribute to the heterogeneity observed across tobacco‐use subgroups. However, the present study did not assess product‐specific exposure levels or physiological effects, and therefore these mechanisms should be interpreted cautiously and examined in future studies.

By contrast, the association between ACEs and OTC drug abuse was not significant among participants who reported illicit drug use. One possible explanation is a ceiling effect, whereby the baseline risk of substance‐related problems in this subgroup may already have been high. However, given the cross‐sectional design, temporal or developmental relationships between OTC drug abuse and illicit drug use cannot be inferred and should be examined in future longitudinal studies.

Mechanistically, our findings are consistent with the self‐medication hypothesis. Individuals with histories of childhood adversity often have chronic dysphoria and impaired stress regulation. They may find that psychoactive compounds provide short‐term relief from painful affective states.^34^ OTC medicines are widely accessible and less stigmatized than controlled substances, which can make them attractive agents for maladaptive coping. Japanese clinical and epidemiologic studies have reported increasing abuse of OTC drugs, especially cough and cold medications. They have also reported psychosocial correlates such as school/home isolation, distress, and weaker parental monitoring among adolescents, as well as psychiatric comorbidity and dependence phenotypes in treatment settings.^18^ These data suggest a pathway through which early adversity contributes to emotional distress and social disconnection. This may lead some individuals to use readily available medicines as a form of self‐directed pharmacological coping.

From a public health perspective, these findings support early identification and trauma‐informed responses within mental health and addiction services. Screening for ACEs may help identify individuals at risk of transitioning from distress to substance abuse. Integrated approaches that consider overlapping behaviors are warranted in Japan, where OTC access is broad, and HTP and e‐cigarette use are common among youth and young adults. The “common liability” perspective suggests that interventions targeting transdiagnostic risk factors (such as impulsivity, emotion dysregulation, and social disconnection) may yield benefits across multiple substance‐related outcomes.^31^

Although educational attainment was not significantly associated with OTC drug abuse after multivariable adjustment, marital status and some income‐related terms showed modest associations. Because these socioeconomic variables were included primarily as adjustment covariates rather than exposures of primary interest, these findings should be interpreted cautiously.^35^ Future studies specifically designed to examine socioeconomic determinants of OTC drug abuse are warranted.

This study has some limitations. First, its cross‐sectional design precludes causal inference, and longitudinal analyses are needed to clarify the temporal sequence. Second, reliance on self‐reporting introduces risks of misclassification and under‐reporting, especially for illicit behaviors. Third, the present analysis could not examine pathways other than substance use behaviors. Factors such as coping style, emotion regulation, or social isolation were not measured, and thus other mechanisms linking ACEs with OTC abuse remain unassessed. Fourth, although the cutoff of four or more ACEs has been widely used in previous epidemiological studies as an indicator of high cumulative adversity, it has not been specifically validated for the Japanese version used in this study. Thus, findings based on the dichotomized ACE variable should be interpreted cautiously. Fifth, the study population was limited to individuals who participated in an online panel survey. Prior methodological research has shown that online survey respondents may differ from the general population in socioeconomic status, health behaviors, and prevalence of substance use (e.g., heavier internet use, higher levels of distress, or greater willingness to disclose stigmatized behaviors).^36^, ^37^ These differences may partially account for the higher prevalence of OTC drug abuse observed in our study compared with existing reports in adolescents (e.g., approximately 1.8% in national data).^12^ Accordingly, the possibility of overestimation should be acknowledged. Future research should adopt prospective designs with repeated measures of adversity, distress, social connectedness, and OTC use. It should also test mechanistic pathways via formal mediation and integrate biomarkers of stress regulation to bridge psychosocial and neurobiological levels of explanation.

In conclusion, ACEs were independently associated with habitual OTC drug abuse in a large Japanese population. The stronger associations observed for HTP and e‐cigarette users and null findings for the illicit drug‐using subgroup are consistent with the clustering of addictive behaviors under a common liability and ceiling effects in high‐risk strata. These observations highlight the long‐term mental health consequences of childhood adversity and support trauma‐informed, transdiagnostic prevention strategies that address emotional distress and social isolation and account for the contemporary landscape of nicotine product use and easy access to psychoactive OTC medications.

## AUTHOR CONTRIBUTIONS

*Conception and design of the study*: Yuhei Mori, Takashi Yoshioka, Takahiro Tabuchi, and Yasuto Kunii. *Data acquisition*: Takahiro Tabuchi. *Data analysis*: Yuhei Mori, Takashi Yoshioka, Takahiro Tabuchi, and Yasuto Kunii. *Drafting of the manuscript and figures*: Yuhei Mori, Takashi Yoshioka, and Yasuto Kunii. All authors reviewed the manuscript and approved its submission.

## CONFLICT OF INTEREST STATEMENT

The authors declare no conflicts of interest.

## ETHICS APPROVAL STATEMENT

The study was approved by the ethics committees of Fukushima Medical University (2025‐002) and Tohoku University Graduate School of Medicine (2024‐1‐673).

## PATIENT CONSENT STATEMENT

All participants provided written informed consent.

## CLINICAL TRIAL REGISTRATION

N/A.

## Supporting information

Supplementary Table S1. Adverse childhood experience items assessed in this study.

Supplementary Table S2. Prevalence of individual ACE items and distribution of total ACE scores.

Supplementary Table S3. Additional analyses treating total ACE score as continuous and ordinal categorical variables.

## Data Availability

The data used in this study are not available in a public repository because they contain personally identifiable information. Based on the regulations for ethical guidelines in Japan, the Research Ethics Committee of the Osaka International Cancer Institute has imposed restrictions on the dissemination of the data collected in this study. All data enquiries should be addressed to the person responsible for data management, Dr. Takahiro Tabuchi, at tabuchitak@gmail.com.
