# Supplementary material for: Association between adverse childhood experiences and over‐the‐counter drug abuse in Japan: A nationwide population‐based cross‐sectional study
Source: PCN Rep. 2026 Jun 2;5(2):e70354. doi: 10.1002/pcn5.70354 (PMC13240047; doi:10.1002/pcn5.70354)
Supplement: Supplementary file 1 — Supplementary Table S1. Adverse childhood experience items assessed in this study. [file PCN5-5-e70354-s002.docx]

Supplementary Table S1. Adverse childhood experience items assessed in this study

| ACE item | Description |
| --- | --- |
| Emotional abuse | Being insulted, humiliated, or made afraid of being physically harmed by a parent or adult in the household |
| Physical abuse | Being pushed, grabbed, slapped, hit, or otherwise physically injured by a parent or adult in the household |
| Sexual abuse | Being sexually touched, asked to sexually touch an adult, or subjected to attempted or completed sexual intercourse |
| Emotional neglect | Feeling unloved, unsupported, or that family members did not care for one another |
| Physical neglect | Lacking adequate food, clothing, protection, or parental care because of intoxication or drug use |
| Parental separation/divorce | Parents being separated or divorced |
| Domestic violence | Mother or stepmother being physically assaulted |
| Household substance misuse | Living with someone who drank heavily, had alcohol-related problems, or misused drugs |
| Household mental illness | Living with someone who had depression, mental illness, or had attempted suicide |
| Household incarceration | Living with someone who had been incarcerated |

ACE, Adverse childhood experience
