# Supplementary material for: Association between adverse childhood experiences and over‐the‐counter drug abuse in Japan: A nationwide population‐based cross‐sectional study
Source: PCN Rep. 2026 Jun 2;5(2):e70354. doi: 10.1002/pcn5.70354 (PMC13240047; doi:10.1002/pcn5.70354)
Supplement: Supplementary file 2 — Supplementary Table S2. Prevalence of individual ACE items and distribution of total ACE scores. [file PCN5-5-e70354-s003.docx]

Supplementary Table S2. Prevalence of individual ACE items and distribution of total ACE scores

1. Prevalence of individual ACE items

| ACE item | n | Prevalence: % (95% CI) |
| --- | --- | --- |
| Parental separation or divorce | 2,627 | 11.5 (10.8–12.2) |
| Emotional abuse | 2,268 | 9.1 (8.5–9.8) |
| Emotional neglect | 2,196 | 8.5 (7.9–9.1) |
| Physical abuse | 1,887 | 7.6 (7.0–8.2) |
| Household mental illness or suicide attempt | 1,300 | 5.1 (4.6–5.5) |
| Witnessing violence against mother or stepmother | 1,212 | 5.0 (4.5–5.5) |
| Sexual abuse | 1,140 | 4.4 (4.0–4.8) |
| Household alcohol or drug misuse | 1,104 | 4.6 (4.1–5.0) |
| Physical neglect | 839 | 3.3 (2.9–3.7) |
| Household incarceration | 432 | 1.8 (1.5–2.1) |

ACE, Adverse childhood experience

1. Distribution of total ACE scores

| Total ACE score | n | Prevalence: % (95% CI) |
| --- | --- | --- |
| 0 | 18,955 | 73.8 (72.8–74.7) |
| 1 | 3,082 | 12.6 (11.8–13.3) |
| 2 | 1,304 | 5.4 (4.9–5.8) |
| 3 | 706 | 2.8 (2.4–3.1) |
| 4 | 569 | 2.4 (2.0–2.8) |
| 5 | 373 | 1.5 (1.2–1.7) |
| 6 | 200 | 0.7 (0.5–0.9) |
| 7 | 117 | 0.4 (0.3–0.5) |
| 8 | 56 | 0.2 (0.1–0.3) |
| 9 | 31 | 0.2 (0.1–0.3) |
| 10 | 31 | 0.2 (0.1–0.4) |

ACE, Adverse childhood experience
