# Supplementary material for: Association between adverse childhood experiences and over‐the‐counter drug abuse in Japan: A nationwide population‐based cross‐sectional study
Source: PCN Rep. 2026 Jun 2;5(2):e70354. doi: 10.1002/pcn5.70354 (PMC13240047; doi:10.1002/pcn5.70354)
Supplement: Supplementary file 3 — Supplementary Table S3. Additional analyses treating total ACE score as continuous and ordinal categorical variables. [file PCN5-5-e70354-s001.docx]

Supplementary Table S3. Additional analyses treating total ACE score as continuous and ordinal categorical variables

A. ACE score as a continuous variable

| Variable | OR | 95% CI | p-value |
| --- | --- | --- | --- |
| ACE score, per 1-point increase | 1.26 | 1.20–1.33 | < 0.001 |
| Sex (Female) | 0.88 | 0.78–0.99 | 0.03 |
| Age | 0.99 | 0.99–1.00 | 0.01 |
| Education (L) | 0.87 | 0.53–1.44 | 0.60 |
| Education (Q) | 0.86 | 0.56–1.32 | 0.49 |
| Education (C) | 0.98 | 0.69–1.39 | 0.91 |
| Education (^4) | 1.04 | 0.83–1.29 | 0.76 |
| Marriage (Never) | 0.76 | 0.66–0.88 | < 0.001 |
| Family (With others) | 1.11 | 0.93–1.33 | 0.25 |
| Alcohol (Yes) | 0.95 | 0.84–1.07 | 0.38 |
| Combustible cigarette (Yes) | 0.99 | 0.84–1.16 | 0.87 |
| Electronic cigarette (Yes) | 3.34 | 2.71–4.11 | < 0.001 |
| Heated tobacco products (Yes) | 2.18 | 1.88–2.53 | < 0.001 |
| Illicit drug (Yes) | 5.72 | 4.52–7.24 | < 0.001 |
| High K6 group (≥13) | 1.46 | 1.27–1.69 | < 0.001 |
| UCLA Loneliness Scale (short-form 3 items) | 1.32 | 1.15–1.51 | < 0.001 |
| Income (L) | 0.80 | 0.67–0.96 | 0.01 |
| Income (Q) | 1.37 | 1.17–1.60 | < 0.001 |
| Income (C) | 0.92 | 0.79–1.07 | 0.29 |
| Income (^4) | 1.23 | 1.07–1.40 | 0.003 |
| Job (No paid work) | 0.74 | 0.64–0.86 | < 0.001 |
| Job (Students) | 1.96 | 0.87–4.40 | 0.10 |

Variable explanations:

ACE: Adverse Childhood Experiences.

SEX: Sex (Female vs. Male).

AGE: Age (continuous).

EDUCATION.L, EDUCATION.Q, EDUCATION.C, EDUCATION^4: Orthogonal polynomial contrasts representing linear (L), quadratic (Q), cubic (C), and quartic trends, respectively, for educational level. The original categories of education were: less than high school or high school, college, undergraduate, graduate and above, and unknown.

MARRIAGE: Marital status (Never vs. Married).

FAMILY: Living arrangement (Lives_with_other vs. Lives_alone).

Alcohol: Alcohol use (Yes vs. No).

K6: Psychological distress (binary; ≥13 vs. ≤12).

UCLA: Loneliness score (binary; ≥ 6 vs. ≤ 5).

INCOME.L, INCOME.Q, INCOME.C, INCOME^4: Orthogonal polynomial terms for income categories allow for the assessment of linear and non-linear trends across ordinal income levels (original categories: <4 million yen, 4–6 million yen, 6–8 million yen, 8–10 million yen, >10 million yen). Specifically, these terms capture whether increases in income are associated with monotonic increases or decreases in the outcome (linear), or more complex patterns such as U-shaped or cubic trends (quadratic or cubic).

JOB: Employment status (Paid_work, No_paid_work, Students).

“Yes” indicates presence or absence of each addictive behavior.

Abbreviations: ACE, adverse childhood experiences; CI, confidence interval; K6, Kessler Psychological Distress Scale; OR, odds ratio; OTC, over-the-counter; UCLA-LS3 (SF-3), UCLA-SF3, three-item short form of the UCLA Loneliness Scale.

B. ACE score as an ordinal categorical variable

| Variable | OR | 95% CI | p-value |
| --- | --- | --- | --- |
| ACE score 0 | 1.00 | Reference | — |
| ACE score 1 | 0.80 | 0.58–1.12 | 0.19 |
| ACE score 2 | 1.58 | 1.09–2.30 | 0.02 |
| ACE score 3 | 2.37 | 1.62–3.48 | <0.001 |
| ACE score 4 | 2.46 | 1.67–3.63 | <0.001 |
| ACE score 5 | 3.27 | 2.19–4.88 | <0.001 |
| ACE score 6 | 3.84 | 2.11–6.99 | <0.001 |
| ACE score 7 | 3.87 | 1.77–8.47 | <0.001 |
| ACE score 8 | 6.72 | 2.73–16.56 | <0.001 |
| ACE score 9 | 14.99 | 3.77–59.55 | <0.001 |
| ACE score 10 | 5.63 | 1.02–31.01 | 0.05 |

In the ordinal categorical analysis, ACE score 0 was used as the reference category. Estimates for the highest ACE scores should be interpreted cautiously because of small cell counts.

Abbreviations: ACE, adverse childhood experiences; CI, confidence interval; OR, odds ratio
